# Conformal leaky-wave antennas for wireless terahertz communications

**DOI:** 10.1038/s44172-023-00067-2

**Published:** 2023-04-19

**Authors:** Hichem Guerboukha, Rabi Shrestha, Joshua Neronha, Zhaoji Fang, Daniel M. Mittleman

**Affiliations:** School of Engineering, Brown University, Providence, Rhode Island 02912 USA

**Keywords:** Electrical and electronic engineering, Applied optics

## Abstract

Future generations of wireless systems are expected to combine the use of high-frequency bands (the terahertz range) with smart interconnected devices (the Internet of Things). To realize this ambitious merging, systems will require antennas that can be mounted on nonplanar objects while generating highly directional beams. Here, we study conformal THz leaky-wave antennas at THz frequencies. We find a rich set of behaviors accessible at THz frequencies dictated by the interplay among the geometrical parameters and the wavelength. We develop simple models to describe the relevant physics, which we verify by an experimental implementation. We also demonstrate data transmission using a conformal THz antenna that can generate multiple high-gain beams with low bit error rates for increased coverage of THz wireless links.

## Abstract (web summary)

Hichem Guerboukha and colleagues describe a conformal (or curved) leaky waveguide in the THz range for antenna applications in wireless communications. The authors describe rich wave behavior at THz frequencies. They also experimentally show application potential for their antennas as multi-beam high-gain transmitters of wireless links with low bit error rates.

## Introduction

As the roll-out of 5G networks continues, many experts are now envisioning future generations of wireless technology^1,2^. These visions generally include a number of important elements, including the use of higher frequency bands above 100 GHz (the terahertz range) for wireless links^3–7^ and the increasing ubiquity of ‘smart’ interconnected devices (the Internet of Things, IoT)^7^. To realize this ambitious combination of ideas, systems will require antennas with a special set of properties. For terahertz (THz) wireless links, antennas will require high gain and high directionality to overcome free-space path loss^8^ while at the same time being able to handle wide bandwidths^9–12^. For widespread ubiquitous deployment, antennas will need to be adaptable to restricted geometries and other physical limitations. At lower frequencies used by legacy wireless systems, this latter requirement has often suggested the use of conformal antennas^13–16^, which (as the name suggests) conform to the shape of a given object with minimal perturbations or extrusions. For example, conformal antennas have played an important role in aerospace communications, where size, weight, and aerodynamic considerations are critical factors^17^. Yet, the longer wavelengths employed by these systems may render the antennas too large to satisfy the size and weight requirements for many IoT devices, and in any event, such systems cannot support the ultrahigh data rates envisioned for future networks. These factors suggest a compelling need for conformal antennas operating in the THz range^18–20^.

**Fig. 1:**
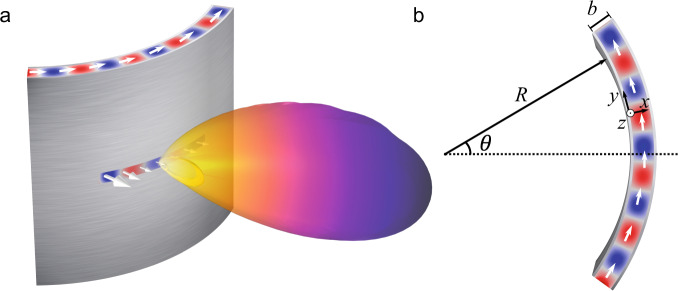
Conformal leaky-wave antenna. **a** The wave propagates in the curved waveguide before leaking through the rectangular aperture. The arrow shows the direction of the propagation. **b** Schematic of the curved waveguide used for the mathematical derivation. Within the antenna, the red and blue colors represent the positive and negative values of the propagating electric field.

Here, we present an original study of conformal THz antennas. Our antenna design builds on the well-known architecture of leaky-wave antennas, as these have proven to be invaluable for numerous applications at both RF frequencies^20–22^ and THz frequencies^23–29^. Moreover, the leaky-wave waveguide offers a variety of possibilities for adaptation to a non-planar geometry^30–34^ and can be easily engineered to explore a wide range of the relevant parameter space. In our implementation, we use an air-filled parallel-plate metal waveguide, which we engineer to conform to a cylindrical geometry, with an azimuthal slot aperture on the exterior surface to enable the guided wave to leak as it propagates around the cylinder (see Fig. 1a). This configuration immediately reveals a rich new set of behaviors accessible to THz devices, dictated by the interplay among three distinct length scales: the wavelength of the guided wave λ_g_ = 2π/β (which is related to the waveguide gap by the requirement that the system operates in the single-mode regime), the radius of curvature *R* of the cylindrical parallel-plate waveguide, and the length of the slot aperture *L* through which guided waves can leak out into free space. We identify three distinct physical regimes associated with the relative values of these three parameters, with unique phenomenology in each case. Unlike earlier situations that have been explored at lower frequencies, these three distinct regimes of operation can all quite feasibly be accessed when operating in the THz range. We explore the relevant physics of each of these regimes and show that our model calculations compare favorably to experimental results. We also show that conformal THz antennas can realistically be used as multi-beam high-gain transmitters by experimental demonstration of wireless links with low bit error rates.

First, we consider an air-filled curved parallel-plate waveguide^35–37^, with the geometry shown in Fig. 1. A version of this configuration can be easily fabricated by bending two metallic sheets that act as the waveguide boundaries. We begin by investigating the effect of curvature on the properties of the fundamental TE mode. We find that the effective refractive index of the guided mode departs from its planar value when the radius of curvature of the waveguide approaches a critical value *R*_*c*_ which increases with frequency. Next, we introduce a narrow slot on the outer metal plate, and we experimentally show how the curvature can affect the directivity of the emitted radiation, even for values of curvature much larger than *R*_*c*_. Finally, we fabricate a conformal multi-beam leaky-wave antenna, and we characterize its performance using a digitally modulated communications signal.

## Results and discussion

### Propagating modes in curved parallel-plate waveguides

We consider the geometry depicted in Fig. 1 consisting of a curved parallel-plate waveguide with a radius of curvature *R*. The waveguide is made of two thin curved metallic plates separated by a distance *b*, with the intervening space filled with a material of refractive index *n*. We consider a transverse electric (TE)-like mode with an electric field polarized perpendicular to the plane of the drawing (*z-axis*) that propagates along the arc length (*y* = *Rθ*), i.e., $\overrightarrow{E}=E(x)\exp (-\gamma y)\hat{z}$, where *γ* = *α* + *jβ* is the complex propagation constant. Under these assumptions, the Helmholtz solution to the wave equation can be written as (see “Methods” for the full derivation)^38,39^:

1 $$\left[{\left(1+\frac{x}{R}\right)}^{2}\frac{{d}^{2}}{d{x}^{2}}+\left(\frac{1}{R}+\frac{x}{{R}^{2}}\right)\frac{d}{dx}+{\left(1+\frac{x}{R}\right)}^{2}{n}^{2}{k}_{0}^{2}\right]E(x)=-{\gamma }^{2}E(x)$$

with ${k}_{0}=2\pi f/c$ is the free space wave vector of frequency *f*. We assume perfect electric boundaries at the metal interfaces: $E(x=0)=E(x=b)=0$. Equation 1 has the form of an eigenequation with the eigenvectors *E*(*x*) and the eigenvalues −*γ*^2^. Using a finite difference scheme, we can discretize the left-hand side of Eq. 1 and write it in a matrix format, which allows us to redefine the problem in terms of finding the eigenvalues of this matrix, which we determine using standard eigensolvers (see “Methods”).

In the following, we assume a waveguide with *b* = 1 mm plate separation filled with air (*n* = 1). As in previous works related to THz leaky-wave antennas^8,23^, we select air as the propagation medium since common dielectrics used in low-frequency leaky-wave antennas exhibit dielectric losses in the THz range^40^. To avoid multimode propagation, we consider frequencies in the single-mode operation band (150–300 GHz). A planar version of this leaky-wave antenna can leak energy across this entire spectrum, making it an ideal candidate for applications requiring large bandwidths^8^. We also mention that our strategy here does not need to rely on the excitation of Floquet modes, as is often the case in periodic leaky-wave antennas filled with a dielectric *n* > 1^22^. Indeed, if we were to use periodic slots instead of a linear slot, more than one leaky mode would be excited, resulting in more than one leaking angle. This fact is a particular consequence of using air-filled waveguides, for which the fundamental mode is always considered a fast-wave mode (*n*_eff_ < 1). This means that it will always leak, and using additional leaky Floquet modes using periodic slots would create additional leaking angles.

**Fig. 2:**
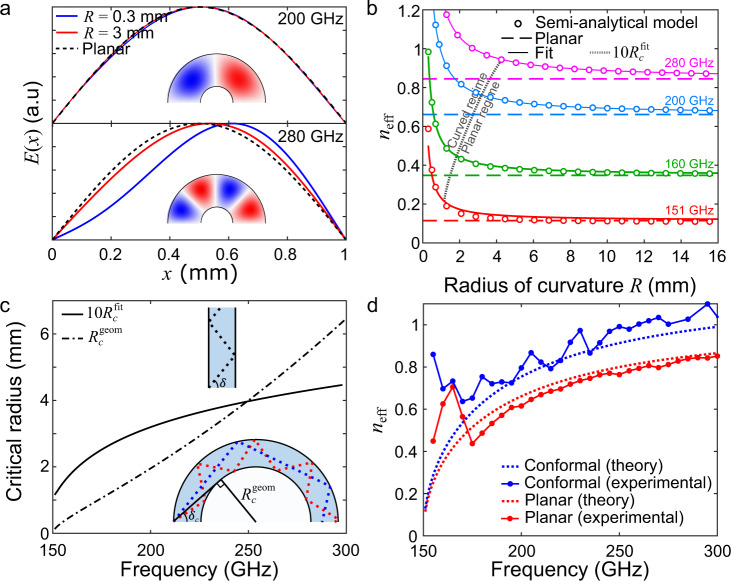
Curved parallel-plate waveguide. **a** Electric field solution (eigenvector) for the fundamental transverse electric (TE_1_)-like mode for 200 GHz (top) and 280 GHz (bottom), for radii of 0.3 mm (blue) and 3 mm (red) compared to a planar parallel-plate waveguide (black dashed). The insets show the electric field propagation obtained with a finite element method simulation with a radius of 0.5 mm for 200 GHz (top) and 280 GHz (bottom) (in red and blue corresponding to positive and negative values of the electric field). **b** Effective refractive index *n*_eff_ as a function of the radius of curvature for various frequencies. The dots are the calculated values obtained from the eigensolution, while the lines correspond to the fits (Eq. 2). The dashed lines correspond to the effective refractive index for a planar parallel-plate waveguide. The gray line is 10*R*_*c*_ and distinguishes the regimes of curved and planar behavior (as explained in the text). **c** Comparison of the critical radii obtained with the fit to the expression obtained from geometrical optics. The critical radius obtained from the fit can be fitted to a fourth-order polynomial: $10{R}_{c}^{{{{{{\rm{fit}}}}}}}[{{{{{\rm{mm}}}}}}]=-9368{f}^{4}+9993{f}^{3}-3983{f}^{2}+715f-45$, with the frequency *f* expressed in units of [THz]. The geometrical optics view of propagation in a parallel-plate waveguide and a curved waveguide are shown in the top and bottom insets, respectively. In the bottom inset, both regimes of alternating reflections (red ray) and whispering-gallery mode reflections (blue ray) are depicted. The black ray corresponds to the critical angle (as explained in the text). **d** Experimentally measured effective refractive index (dots) in the single-mode operation range (150–300 GHz, *b* = 1 mm) compared to the analytical models (bold lines) for a conformal waveguide with *R* = 3.5 mm (blue) and a planar waveguide (red).

Among the solutions found, only the TE_1_-like modes are associated with purely imaginary propagation constants, while for higher-order modes, the propagation constants are purely real. This means that only the TE_1_-like modes can propagate, which is consistent with the fact that the chosen frequencies lie in the single-mode operation band of a standard parallel-plate waveguide with *b* = 1 mm. Figure 2a shows the electric field distribution of the fundamental TE_1_-like mode for a radius of curvature of 0.3 and 3 mm at frequencies of 200 and 280 GHz. For 200 GHz, the electric field distributions are similar to that of a parallel-plate waveguide (dashed line), while for 280 GHz, the mode is deformed for the smaller radius with energy shifted toward the outer plate (located at *x* = 1 mm).

We can also compute the effective refractive index (derived from the eigenvalue) as a function of the curvature *R* (colored dots in Fig. 2b). We found that the results fit very well to the following functional form (bold lines in Fig. 2b):

2 $${n}_{{{{{{\rm{eff}}}}}}}(R)={n}_{{{{{{\rm{eff}}}}}}}^{{{{{{\rm{planar}}}}}}}+\frac{{R}_{c}^{{{{{{\rm{fit}}}}}}}}{R}$$

Here, ${n}_{{{{{{\rm{eff}}}}}}}^{{{{{{\rm{planar}}}}}}}$ is the well-known result for the fundamental TE_1_ mode of an air-filled planar plate waveguide^8^ (shown as dotted horizontal lines in Fig. 2b):

3 $${n}_{{{{{{\rm{eff}}}}}}}^{{{{{{\rm{planar}}}}}}}=\sqrt{1-{\left(\frac{{f}_{0}}{f}\right)}^{2}}$$

where *f*_0_ = *c*/2*b* is the cutoff frequency. As one would expect, the effective refractive index asymptotically approaches ${n}_{{{{{{\rm{eff}}}}}}}^{{{{{{\rm{planar}}}}}}}$ when the radius increases (*R* → ∞).

In Eq. 2, ${R}_{c}^{{{{{{\rm{fit}}}}}}}$ is a fitting parameter, which we found to increase with frequency (Fig. 2c). It specifies a radius below which the effective index differs substantially from the planar value. We can consider that a curvature *R* small enough so that ${R}_{c}^{{{{{{\rm{fit}}}}}}}/R \, > \, 0.1$represents a significant change of >0.1 in the effective mode index. Therefore, the value of $10{R}_{c}^{{{{{{\rm{fit}}}}}}}$ can be seen as a critical radius dividing the regimes of planar-like behavior and curved behavior (shown as a dashed gray line in Fig. 2b).

To understand why this critical radius increases with frequency, we consider the perspective of geometrical optics shown in the inset of Fig. 2c. A TE_1_ mode propagating inside a parallel-plate waveguide can be viewed as the superposition of two plane waves alternatively reflecting from one plate to the other. The angle of reflection derives from the wavevector and depends on the frequency: $\cos \delta ={f}_{0}/f$^41^. Now, consider a curved section of radius *R*. Depending on the reflected angle and the relative values of *R* and *b*, two possibilities can arise. In the first one, the ray reflecting from the outer plate continues propagating along the arc by alternately reflecting between the inner and outer plates (red dotted ray in the inset of Fig. 2c). This mechanism happens for large *R* (or small *δ*) is similar to the propagation in a parallel-plate waveguide. However, a second possibility can occur. When *R* is small enough (or large *δ*), the ray originating from the outer plate can keep propagating by reflecting only from the outer plate, without ever bouncing off the inner plate (blue dotted ray in the inset of Fig. 2c). This happens when the radius of curvature falls below a critical radius that can be geometrically evaluated based on the critical angle: $\cos {\delta }_{c}=\frac{\sqrt{{(R+b)}^{2}-{R}^{2}}}{R+b}$. We can equate that angle to that of a TE_1_ mode ($\cos \delta ={f}_{0}/f$) to predict a frequency-dependent critical radius under this geometrical optics interpretation:

4 $${R}_{c}^{{{{{{\rm{geom}}}}}}}=b{\left(\frac{f}{{f}_{0}}\right)}^{2}\left[1-{\left(\frac{{f}_{0}}{f}\right)}^{2}+\sqrt{1-{\left(\frac{{f}_{0}}{f}\right)}^{2}}\right]\approx \frac{3b}{2}\left[\frac{4}{3}{\left(\frac{f}{{f}_{0}}\right)}^{2}-1\right]$$

where the approximation becomes more valid when $\nu \gg {\nu }_{0}$. Figure 2c shows both critical radii obtained from the fit (bold line) and from this geometrical optics interpretation (dashed line). The simplified geometrical optics model matches the general trend observed from fitting to the eigenmode solutions, suggesting that it captures the essence of the underlying physics. Of course, ray optics is only a low-order approximation here, so perfect agreement is not expected. We note that the regime of propagation by reflection from the outer plate is similar to that of a whispering-gallery mode^42^. In fact, if *R* is small enough ($R\ll b$) such that the terms in *R*^−2^ dominate over the other terms in Eq. 1, then this equation can be reduced to the Bessel equation used to describe the two-dimensional whispering-gallery mode with a single curved reflective surface^43,44^. This is further confirmed by the appearance of the electric field distribution for small *R* (bottom of Fig. 2a), which resembles that of a whispering gallery mode. Overall, these results suggest that depending on the value of *R*, two extreme regimes can exist: (1) a planar TE_1_-like mode for large *R* and (2) a whispering-gallery-like mode for small *R*. In general, for intermediate *R*, we can expect the supported mode to be a combination of both these regimes. It is remarkable that these regimes can all be accessed at THz frequencies with relative ease.

To confirm the prediction of our semi-analytical model, we perform an experimental characterization of curved waveguides using the cut-back technique^45,46^. With a THz time-domain spectroscopy system in transmission geometry^47^, we measure the propagation of a THz pulse through two lengths of the same waveguide and then calculate the phase of the Fourier transform of the measured time-domain pulses. By subtracting the phases from the two lengths of waveguide Δ*ϕ*, we can obtain an experimental evaluation of the effective refractive index:

5 $${n}_{{{{{{\rm{eff}}}}}}}=\frac{c}{2\pi f\Delta L}\Delta \phi +1$$

where Δ*L* is the length difference between the two waveguides.

In Fig. 2d, we compare the measured refractive index of a curved waveguide (*R* = 3.5 mm, blue dots) to that of a planar waveguide (red dots) across the single-mode operation band for *b* = 1 mm. The experimental points agree well with the semi-analytical model for a curved waveguide (for *R* = 3.5 mm, red curve) and with the analytical result for a planar waveguide (Eq. 3, blue curve).

The important difference between these two results (curved vs. planar) suggests that the distortion of the guided mode induced by the curvature may also have an impact on the waveguide dispersion. An important change in waveguide dispersion can have implications for its use in a communications context, as it can lead to changes in the inter-symbol interference caused by group delay dispersion^48^. The dispersion is typically quantified using a Taylor expansion of the propagation constant, and the second-order term is defined as the group velocity dispersion (GVD):

6 $${{{{{\rm{GVD}}}}}}=\frac{{d}^{2}\beta }{d{\omega }^{2}}=\frac{2}{c}\frac{d{n}_{{{{{{\rm{eff}}}}}}}}{d\omega }+\frac{\omega }{c}\frac{{d}^{2}{n}_{{{{{{\rm{eff}}}}}}}}{d{\omega }^{2}}$$

**Fig. 3:**
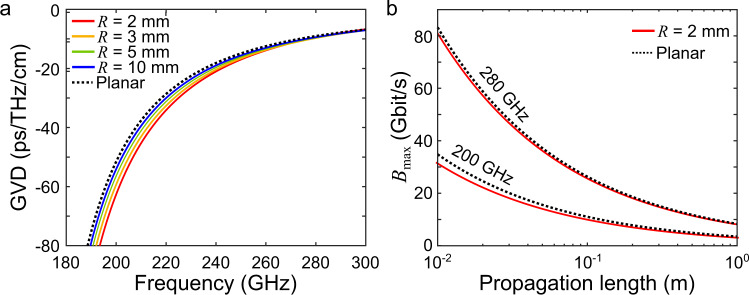
Effect of the curvature on dispersion. **a** Group-velocity dispersion (GVD) for curved waveguides (color lines) and for a planar waveguide (dotted line). **b** Achievable maximal achievable bitrate *B*_max_ (when limited by dispersion) for frequencies of 200 and 280 GHz when comparing the extreme cases of a planar waveguide and a small radius of 2 mm.

Figure 3a shows the value of the GVD as a function of frequency for various radii of curvature. Here, the GVD is expressed in ${{{{{\rm{ps}}}}}}\cdot {{{{{{\rm{THz}}}}}}}^{-1}\cdot {{{{{{\rm{cm}}}}}}}^{-1}$, and corresponds to ps of time broadening at a given THz frequency and for a given waveguide length in cm^40,48^. As before, when the radius of curvature increases, the GVD approaches the analytical value of the GVD for a planar leaky-wave antenna that can be derived from Eq. 3:

7 $${{{{{{\rm{GVD}}}}}}}^{{{{{{\rm{planar}}}}}}}=-\frac{b}{\pi {c}^{2}}\frac{1}{{\left[{\left(\frac{f}{{f}_{0}}\right)}^{2}-1\right]}^{3/2}}$$

This calculation shows that the radius of curvature has a larger effect at lower frequencies than at higher frequencies. For example, at 200 GHz, the GVD is $-49\,{{{{{\rm{ps}}}}}}\cdot {{{{{{\rm{THz}}}}}}}^{-1}\cdot {{{{{{\rm{cm}}}}}}}^{-1}$ for a planar parallel-plate waveguide and changes to $-60\,{{{{{\rm{ps}}}}}}\cdot {{{{{{\rm{THz}}}}}}}^{-1}\cdot {{{{{{\rm{cm}}}}}}}^{-1}$ for a small radius of curvature of 2 mm, while it changes from $-\,9.5\,{{{{{\rm{ps}}}}}}\cdot {{{{{{\rm{THz}}}}}}}^{-1}\cdot {{{{{{\rm{cm}}}}}}}^{-1}$ to $-\,9\,{{{{{\rm{ps}}}}}}\cdot {{{{{{\rm{THz}}}}}}}^{-1}\cdot {{{{{{\rm{cm}}}}}}}^{-1}$ at 280 GHz. These considerations indicate that waveguides with small values of *R* will support lower data rates. For example, assuming amplitude shift keying (ASK) modulation, the maximal bitrate *B*_max_ (when GVD is the only limiting factor) is given by^49^:

8 $${B}_{{{\max }}}=\frac{1}{4\sqrt{|{{{{{\rm{GVD}}}}}}|l}}$$

This is plotted as a function of propagation length *l* in Fig. 3b for the two cases mentioned above (*R*→∞ and *R* = 2 mm). At the lower frequency (200 GHz), a propagation length of 1 cm leads to a ~11% decrease in the supported maximal bit rate, whereas the decrease amounts to only ~3.6% at the higher frequency (280 GHz). Overall, these results indicate that the increase (in magnitude) of the dispersion results in a few % reduction of the maximal bitrate when comparing the extreme cases of planar and a radius of 2 mm. This suggests that the waveguide dispersion is mainly driven by the dispersion characteristics of the TE_1_ mode of the planar parallel-plate waveguide, which is known to increase (in magnitude) near the cutoff frequency^41^. In any case, it would be possible to compensate for some of this dispersion by introducing dispersion of the opposite sign, for example, with a Bragg waveguide^50^.

### Radiation patterns from conformal leaky-wave antennas

We now turn to the analysis of the far-field radiation from a leaky-wave antenna based on the curved parallel-plate waveguide described above. Based on those results, one would not be surprised that the radiation pattern is significantly modified by the curvature when *R* is small enough to induce a significant change in the effective index (see Fig. 2b). However, a different physical effect produces substantial changes to the far-field radiation pattern even at much larger values of *R*, resulting from the fact that the wave vector of the guided wave changes its angle as the mode propagates. If this angle changes significantly within the arc length *L* defined by the length of the slot aperture through which radiation couples out into free space, the far-field radiation pattern is substantially impacted.

As shown in Fig. 1, we may introduce a narrow slot in the outer plate, with its axis along the propagation direction of the guided wave (azimuthally, as the *y*-axis in Fig. 1b). The radiated electric field ${\overrightarrow{E}}^{{{{{{\rm{rad}}}}}}}$ in the far-field can be computed from the near-field response using the Stratton–Chu diffraction integral^51,52^:

9 $${\overrightarrow{E}}^{{{{{{\rm{rad}}}}}}}(\phi )=\frac{jk}{4\pi }{\overrightarrow{r}}_{\phi }\times \int [\overrightarrow{n}\times \overrightarrow{E}]\exp (\,jk\,{\overrightarrow{r}}_{\theta }\cdot {\overrightarrow{r}}_{\phi })dS$$

**Fig. 4:**
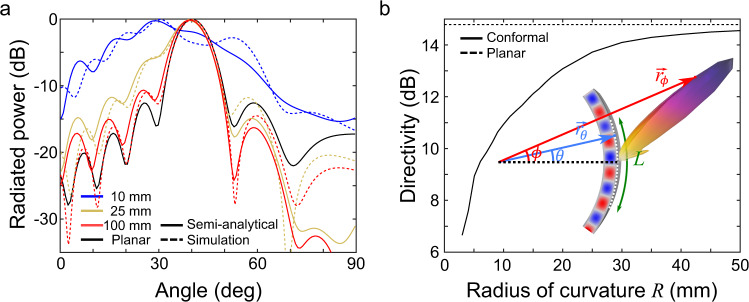
Radiation patterns emitted from the conformal leaky-wave antenna. **a** Far-field radiation pattern obtained with the semi-analytical model (Eq. 9) for curved leaky-wave antennas (color lines) compared to finite element method simulations (dashed color lines) and to an analytical planar leaky-wave antenna (bold black, Eq. 14). The frequency is 200 GHz, and we assume *α* = 100 m^−1^ and *L* = 10 mm. **b** Directivity as a function of the radius of curvature for a conformal (bold line) and a planar leaky-wave antenna (dashed line). These results were obtained with a finite element method simulation. Inset shows the variables used in calculating the far-field integral in Eq. 9. Red and blue shadings represent the positive and negative values of the electric field.

Here, ${\overrightarrow{E}}^{{{{{{\rm{rad}}}}}}}(\phi )$ is the radiated electric field, while $\overrightarrow{E}$ is the electric field directly under the aperture slot. The vector ${\overrightarrow{r}}_{\phi }=(\cos \phi ,\,\sin \phi ,0)$ is the unit vector pointing from the origin to the far-field point, $\overrightarrow{n}=(\cos \theta ,\,\sin \theta ,0)$ is the unit vector normal to the cylindrical slot, and ${\overrightarrow{r}}_{\theta }=R\overrightarrow{n}=R(\cos \theta ,\,\sin \theta ,0)$ is the radius vector of the aperture slot (see inset of Fig. 4b for a schematic).

If the slot is narrow relative to the wavelength, the electric field under the slot, polarized in the *z*-direction, can be described as a harmonic wave with an exponentially decaying amplitude^9^:

10 $$\overrightarrow{E}(\theta )=\exp [-j(\beta +j\alpha )(R+b)\theta ]\hat{z}$$

where *β* is the wavevector of the mode propagating inside the curved waveguide as computed above. The parameter *α* is the leakage rate and denotes the radiation losses of the propagating mode. It can generally be derived from the characteristics of the aperture perturbation^53,54^. For example, for a rectangular slot, a large width results in most of the energy leaking at the beginning of the slot and therefore corresponds to a large *α*. In general, the constant *α* is chosen so that most of the energy leaks before it reaches the end of the antenna, with the residual being absorbed by a load to avoid back-reflection. In the following experiments, we select slot widths of 2 mm, which were found experimentally to allow most of the energy to leak. If the aperture is sufficiently narrow, the electric field is uniform in the transverse direction (*z*-direction in Fig. 1b), which means that the surface integral over the slot can be simplified to a one-dimensional integral over the angular opening of the aperture (−*L*/2*R* to *L*/2*R*), where we assume the slot to be centered at *θ* *=* 0. With these approximations, Eq. 9 can be simplified to:

11 $${\overrightarrow{E}}^{{{{{{\rm{rad}}}}}}}(\phi )=-\frac{jk}{4\pi }[\sin \phi \,{S}_{1}-\,\cos \phi \,{S}_{2}]\hat{z}$$

where *S*_1_ ($\phi$) and *S*_2_ ($\phi$) are the numerical solutions of the following integrals:

12 $${S}_{1}(\phi )={\int }_{-L/2R}^{L/2R}\,\sin \theta \exp (-j(\beta +j\alpha )(R+b)\theta )\exp [\,jkR\,\cos (\theta -\phi )]d\theta$$

and

13 $${S}_{2}(\phi )={\int }_{-L/2R}^{L/2R}\,\cos \theta \exp (-j(\beta +j\alpha )(R+b)\theta )\exp [\,jkR\,\cos (\theta -\phi )]d\theta$$

where the integrals over *θ* correspond to integration over the aperture.

Figure 4a shows various radiation patterns for radii of curvature of 10 mm (blue), 25 mm (brown), and 100 mm (red) as calculated using Eq. 9 (bold color lines). As can be seen, our semi-analytical model agrees well with the results obtained using a numerical finite-element method solver (dashed). As one would expect, for a large value of *R*, the radiation pattern is similar to the analytical results for a planar leaky-wave antenna (shown by the black solid curve)^55,56^:

14 $$|{\overrightarrow{E}}^{{{{{{\rm{rad}}}}}}}(\phi )|=L{{{{{\rm{sinc}}}}}}\left[(\beta -{k}_{0}\,\sin \phi -j\alpha )\frac{L}{2}\right]$$

We emphasize, however, the significant broadening of the radiation pattern even for a value of *R* as large as 25 mm, which is off the scale to the right in Fig. 1b. In other words, this notable change in directivity is not a result of the distortion of the guided mode and the associated change in effective refractive index detailed in Fig. 1b. Rather, it is a direct consequence of the fact that the wave vector turns as the mode propagates. Figure 4b shows the computed directivity vs. *R* at 200 GHz for a fixed value of *L* = 10 mm. Clearly, as the radius increases, the directivity asymptotically approaches that of a planar leaky-wave antenna. However, for smaller radii, for which the ratio *L*/*2*π*R* becomes significant (but for which *R* is not yet small enough to approach *R*_*c*_ λ_g_), we find a regime of operation in which the antenna’s directivity is impacted despite no significant change in the effective index of the guided mode.

Equation 14 also reveals another important aspect of the radiation pattern of a leaky-wave antenna, which is that the radiation peaks at an angle defined by the phase-matching condition: $\cos \phi =\beta /{k}_{0}$. Specifically, this means that different frequencies peak at different angular locations, producing a rainbow-like radiation pattern. For a planar leaky-wave antenna based on parallel plates, the frequencies peak according to the relation $\sin \phi =c/2bf$. This angular dispersion can lead to some issues when transmitting large bandwidths since different spectral components will propagate at slightly different angles, thus causing an angular spreading of the information content^57^. This issue becomes more apparent when using a high-gain antenna with an angular narrowing of their main lobe and thus less overlap from the different spectral components. Therefore, in general, characteristics of the angular dispersion become an additional design constraint when engineering an antenna for a particular application.

**Fig. 5:**
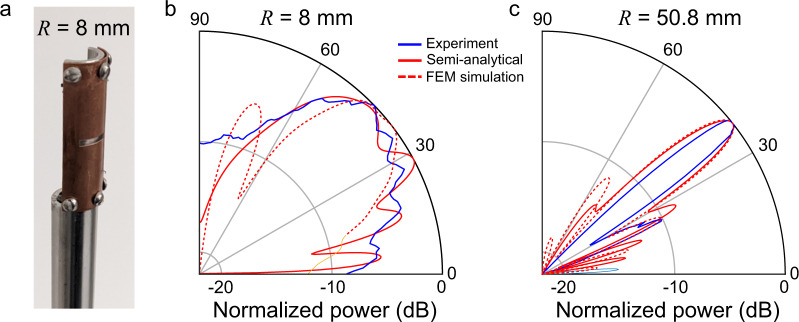
Effect of the curvature on the radiation pattern. **a** Photograph of the fabricated conformal leaky-wave antenna with *R* = 8 mm. **b** Experimentally measured radiation pattern (bold blue) compared to the semi-analytical model (bold red) and the finite element method simulation (dashed red) for the conformal antenna with a radius of curvature of 8 mm and **c** 50. 8 mm. The finite element method simulations show directivities of 14 dB and 18 dB for radii of 8 mm and 50.8 mm, respectively.

For experimental verification, we fabricate two conformal leaky-wave antennas with radii of curvature of 8 mm and 50.8 mm. In both waveguides, a 2-mm wide, 10-mm long slot is cut in a flexible copper sheet and placed on top of a cylindrical aluminum tube (Fig. 5a shows the smaller *R* = 8 mm device). A 1-mm spacer is placed between the two metal plates to ensure a constant plate separation throughout the length of the antenna. The TE1-like mode is excited using a 200 GHz source obtained from a frequency-multiplier source, and the radiation pattern is measured in the far field with a Schottky diode mounted on a rotating rail^58^ (see “Methods” for details). Figure 5b, c show the measured normalized radiation patterns for the antenna of small and large radius of curvature, respectively (blue curves), which match well with the predictions of the semi-analytical model (red solid curves) and the finite-element method simulations (red dashed curves). These results clearly show how the radius of curvature affects the directivity; for the smaller radius, the beam is wider (low directivity), while it becomes narrower for the large radius (high directivity).

The reduction of directivity for decreasing the radius of curvature can be countered to some extent. Indeed, as mentioned, because the wave leaks at an angle specified by the wavevector, if the latter changes as the wave propagates, the outgoing angle changes as well. Therefore, one way to counter this loss of directivity is to design a leaky-wave antenna in which the wavevector changes as a function of the propagating length. For example, the strategy implemented in ref. ^34^ consisted of changing the periodicity of the periodic leaky-wave antenna to affect the wavevector of the outgoing Floquet mode. As mentioned before, in the case of an air-filled leaky-wave antenna, using periodic slots would result in more than one excited leaky mode and, therefore, more than one outgoing angle. A strategy compatible with our geometry would be to change the plate separation *b*. Indeed, as shown in Eq. 3, this would result in a change of the effective index and, therefore, in a change of outgoing angle. This strategy has some limits since a large plate separation can also result in multimode propagation, which is preferable to avoid. Also, a more careful analysis of the leakage is necessary to ensure a uniform power contribution across the slot length. Engineering of the leakage rate can be achieved using a tapered slot geometry, as shown in ref. ^30^.

**Fig. 6:**
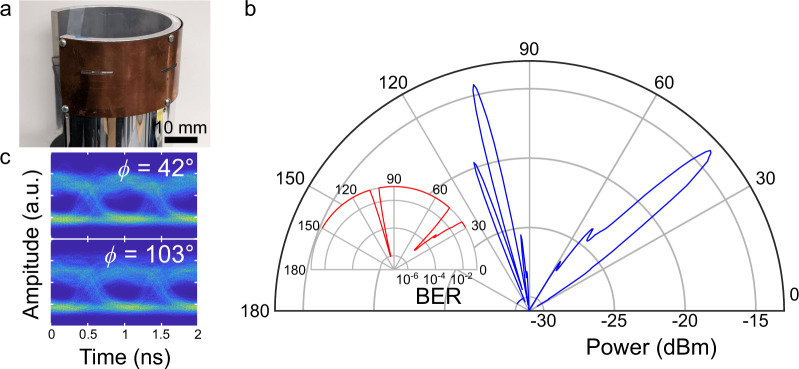
Multi-beam leaky-wave antenna. **a** Photograph of the multibeam conformal leaky-wave antenna. **b** Radiation pattern for the conformal multi-beam antenna (blue). Inset shows the corresponding measured bit error rates (red). **c** Eye diagrams for the main peaks.

Finally, we demonstrate a proof-of-concept multibeam conformal antenna. At THz frequencies, highly directional beams are necessary to account for the strong free-space path loss. This requirement restricts the coverage of an antenna to a small range of angles. Thus, there is growing interest in the use of multi-beam antennas for greater angular coverage^59,60^. Here, we incorporate two different slot apertures located at two different azimuthal positions. We perform these experiments using a modulated data stream to accurately characterize the performance of the device in the context of wireless communications. We use the on-off keying (OOK) modulation scheme, which is specified in the recent IEEE 802.14.3e standard to be the preferred physical layer mode for low-complexity devices as those intended to be used in IoT applications^61^. As before, the conformal multi-beam antenna is fabricated with a flexible copper sheet placed on top of a 50.8-mm radius aluminum tube (Fig. 6a). This time, two 2-mm wide, 20-mm long slots are cut in the copper sheet. The slots are angularly separated by 60° and parallel but laterally separated by 2 mm. The excitation source is focused on the input end of the antenna at a vertical position to excite the waveguide at the positions of the two slots. This ensures that part of the guided wave’s energy exits through the first slot while the rest continues inside the waveguide until it reaches the second slot.

Figure 6b shows the measured radiated pattern, demonstrating the generation of two directional beams of similar amplitudes. As designed, the peaks are angularly separated by 60°. We emphasize that this conformal antenna can provide coverage exceeding 90° of angular range, which contrasts with that which can be obtained using a planar leaky-wave antenna with a single slot^9^. When sending a 1 Gbit/s data stream (OOK), we achieve bit error rates smaller than 10^−5^ for both of the radiated peaks (inset of Fig. 6b), confirming the practicality of this antenna for communication links. The measured eye diagrams (Fig. 6c) show clear eye openings and little distortions and jitter. As anticipated from Fig. 3 above, there is little effect of inter-symbol interference due to waveguide dispersion^62^, even for the peak emitted from the second slot.

## Conclusion

In conclusion, we have studied and demonstrated conformal leaky-wave antennas at THz frequencies. We developed a semi-analytical model to describe the effect of the curvature on the propagation of the TE_1_-like mode in a parallel-plate waveguide. We were able to distinguish two regimes of propagation (curved and planar), and we found that when the curvature is large enough (1 cm or more), the mode propagates as if it was propagating in a flat parallel-plate waveguide. Conversely, when the curvature is very small, we observed that the mode converts into a whispering-gallery-like mode. We also studied the impact of the curvature on the waveguide’s dispersion, and we noted that the dispersion was more affected by tight bending and at lower frequencies. Then, we allowed the propagating mode to leak in free space by adding a narrow rectangular slot on the outer metallic plate. We found that the far-field radiation pattern is importantly modified even for large values of curvature, and we attributed this phenomenon to the fact that the wave vector changes its angle as the mode propagates. If the angle is sufficiently varied within the arc length defined by the aperture geometry, the directivity of the emitted radiation decreases. Finally, we demonstrated a conformal multi-beam antenna with two apertures located at two different azimuthal positions, and we showed that it was able to transmit two peaks of similar amplitude with bit error rates <10^−5^. By shedding new light on the physics of curved waveguides, we hope our findings can accelerate the ubiquitous implementation of THz antennas for future wireless communications systems and IoT applications.

## Methods

### Derivation and discretization of the Eigenequation

The Helmholtz solution to the wave equation is

A1 $${\nabla }^{2}\overrightarrow{E}=-{n}^{2}{k}_{0}^{2}\,\overrightarrow{E}$$

We consider polar coordinates, in which the Laplace operator can be written as:

A2 $${\nabla }^{2}=\frac{1}{r}\frac{\partial }{\partial r}\left(r\frac{\partial }{\partial r}\right)+\frac{1}{{r}^{2}}\frac{\partial }{\partial \theta }$$

As a solution, we consider a propagation along the arc length *y* = *rθ*, and we replace the radial coordinate with the value at the inner radius of the waveguide, i.e., *x* = *r* − *R*. Considering a TE-like mode with an electric field polarized perpendicular to the plane of the drawing (*z*-axis), the electric field can be written as:

A3 $$\overrightarrow{E}(x,y)=E(x)\exp (-\gamma y)\hat{z}$$

where *γ* = *α* + *jβ* is the complex propagation constant. Under these assumptions, the Laplace operator becomes:

A4 $${\nabla }^{2}=\frac{{\partial }^{2}}{\partial {x}^{2}}+\frac{1}{x+R}\frac{\partial }{\partial x}+\frac{{R}^{2}}{{(x+R)}^{2}}\frac{{\partial }^{2}}{\partial {y}^{2}}$$

Then, the Helmholtz solution becomes a 1D equation on *x*:

A5 $$\frac{{d}^{2}E(x)}{d{x}^{2}}+\frac{1}{x+R}\frac{dE(x)}{dx}+{n}^{2}{k}_{0}^{2}E(x)=-\frac{{R}^{2}}{{(x+R)}^{2}}{\gamma }^{2}E(x)$$

which can be rearranged to yield the result of Eq. 1. We note that Eq. A5 is a simple unidimensional equation over the variable *x*, which corresponds to the coordinate between the plates. Because our model natively derives from the case of a planar parallel-plate waveguide, it allows us to naturally make connections with the parallel-plate waveguide and therefore obtain a simple understanding of the physical phenomenology. Similar advanced modeling using a cylindrical harmonics formulation could also be used in more complex geometries.

It is worth mentioning that when *R* → ∞, Eq. 1 reduces to the Helmholtz equation in cartesian coordinates, i.e., with ${\nabla }^{2}={d}^{2}/d{x}^{2}$:

A6 $$\frac{{d}^{2}E(x)}{d{x}^{2}}+{n}^{2}{k}_{0}^{2}E(x)=-{\gamma }^{2}E(x)$$

Furthermore, we note that Eq. 1 was proposed in previous works such as ref. ^38^. However, those works dealt with large radii compared to the waveguide size *b*. Thus, the terms in *R*^−2^ were neglected. Here, we include the cases when *R* < *b* (which can be easily fabricated at THz frequencies); therefore, we kept the terms in *R*^−2^. Our model contains no approximation of the radius of curvature and is therefore applicable to a wide range of radii.

To numerically solve eigenequation Eq. 1, we consider a discretization of the *x*-axis with discrete steps *h*. The discretized first-order derivative is

A7 $$\frac{dE}{dx}=\frac{{E}_{i+1}-{E}_{i-1}}{2h}+O({h}^{2})$$

and the discretized second-order derivative is

A8 $$\frac{{d}^{2}E}{d{x}^{2}}=\frac{{E}_{i+1}-2{E}_{i}+{E}_{i-1}}{{h}^{2}}+O({h}^{2})$$

Then, the discretized eigenequation becomes:

A9 $$	{E}_{i}\left\{-\frac{2}{{h}^{2}}{\left(1+\frac{ih}{R}\right)}^{2}+{n}^{2}{k}_{0}^{2}\,{\left(1+\frac{ih}{R}\right)}^{2}\right\}+{E}_{i-1}\left\{\frac{1}{{h}^{2}}{\left(1+\frac{ih}{R}\right)}^{2}\right.\\ 	 \left.-\frac{1}{R}\,\left(1+\frac{ih}{R}\right)\frac{1}{2h}\right\}+{E}_{i+1}\left\{\frac{1}{{h}^{2}}{\left(1+\frac{ih}{R}\right)}^{2}+\frac{1}{R}\,\left(1+\frac{ih}{R}\right)\frac{1}{2h}\right\}=-{\gamma }^{2}{E}_{j}$$

Left-side of Eq. A9 is a linear system of equations, which can be written as a tridiagonal matrix, where the first, second, and third term corresponds to the main diagonal, the diagonal below, and the diagonal above, respectively.

### Radiation patterns and communications experiments

The radiation patterns are measured using a 200 GHz source made of a frequency-multiplier chain with a multiplication factor of 16 and driven by an RF oscillator outputting 12.5 GHz. The radiation is first generated in free space using a horn antenna before being collected by a biconvex dielectric lens and focused into the tapered input of the curved waveguide^63^. The tapered input allows near-unity efficiency in this air-to-air coupling strategy^58^. To avoid this free-space coupling, an alternative strategy would be to replace the horn antenna on the transmitter side and directly couple the curved antenna to the output of the rectangular waveguide. The radiation pattern is measured in the far field with a Schottky diode mounted on a rotating rail.

For the communications experiments, the 12.5 GHz single-tone is modulated with a pulse pattern generator outputting 1.12 Gbps OOK signal with a pseudo-random binary sequence of length 2^7^–1. The signal is received with a zero-bias Schottky diode before passing through low-pass filters to obtain the baseband signal between 0.1 and 6 GHz. The signal is then routed to a power meter, a real-time bit error rate (BER) tester, or an oscilloscope to provide eye diagrams.

## Data Availability

The data that support the findings of this study are available from the corresponding author upon reasonable request.
